# When Treatment Becomes the Trigger: Fludrocortisone-Associated Hypokalaemia in an Older Adult

**DOI:** 10.7759/cureus.112116

**Published:** 2026-07-05

**Authors:** Khaing Cherry Phyo, Zambu Kyaw, Zin Mar Pyone, Lin Pyae Phyo Aung, Thyn Thyn

**Affiliations:** 1 Emergency Department, Barnsley Hospital NHS Foundation Trust, Barnsley, GBR; 2 General Internal Medicine Department, Barnsley Hospital NHS Foundation Trust, Barnsley, GBR; 3 Diabetes and Endocrinology Department, Barnsley Hospital NHS Foundation Trust, Barnsley, GBR; 4 Geriatrics Department, Barnsley Hospital NHS Foundation Trust, Barnsley, GBR

**Keywords:** fludrocortisone, hypokalaemia, medication-induced electrolyte disturbances, older adults/geriatrics, postural hypotension

## Abstract

Hypokalaemia is a common electrolyte abnormality in older adults and is frequently multifactorial in origin. Although often mild and asymptomatic, severe hypokalaemia can result in significant neuromuscular and cardiac complications. Medication-induced hypokalaemia remains an important and potentially reversible cause, particularly in frail elderly populations. We present the case of an 85-year-old woman who presented with generalised weakness, muscle cramps, reduced mobility, and a fall, and was found to have significant hypokalaemia in the context of long-term fludrocortisone therapy prescribed for postural hypotension. Following intravenous potassium replacement and discontinuation of fludrocortisone, with initiation of midodrine as an alternative therapy, the patient’s potassium levels normalised and her symptoms improved. This case highlights the importance of medication review and regular electrolyte monitoring in older adults, particularly those receiving mineralocorticoid therapy.

## Introduction

Hypokalaemia, defined as a serum potassium concentration below approximately 3.5 mmol/L, is one of the most common electrolyte disturbances seen in clinical practice and can affect people of all ages, including older adults [1]. In many patients, hypokalaemia is mild and asymptomatic; however, severe hypokalaemia may result in clinically significant complications, including muscle weakness, neuromuscular dysfunction, and cardiac arrhythmias [2]. Rare neuropsychiatric manifestations, including psychosis, delirium, agitation, and hallucinations, have been reported in association with hypokalaemia, although these are uncommon and described in case reports and small clinical observations [3]. Older adults are particularly vulnerable to electrolyte disturbances due to age-related changes in renal function, polypharmacy, and the presence of multiple comorbidities [4].

Fludrocortisone, a synthetic mineralocorticoid, is occasionally prescribed for the management of orthostatic hypotension in older adults [5]. Its mechanism of action involves sodium retention and increased renal potassium excretion, which can predispose patients to hypokalaemia, particularly with prolonged use. Regular monitoring of serum electrolytes is therefore recommended during ongoing fludrocortisone therapy, especially in older patients who may be more susceptible to adverse effects [5].

Hypokalaemia in older individuals may present with non-specific or atypical symptoms, including weakness, falls, and changes in mental state, which can easily be misattributed to other common geriatric syndromes. In the context of fludrocortisone therapy for orthostatic hypotension, hypokalaemia is a recognised adverse effect due to increased renal potassium excretion, and regular monitoring of serum electrolytes is recommended during treatment [6]. Recognising its diverse presentations and reviewing contributing medications are vital steps in the holistic assessment of frail older adults. Although fludrocortisone-associated hypokalaemia is a recognised adverse effect, this case highlights how its presentation may be easily overlooked in older adults because of its non-specific clinical features. It emphasises the importance of careful medication review and regular electrolyte monitoring to facilitate early recognition and prevent avoidable morbidity.

## Case presentation

An 85-year-old female presented to the Emergency Department following referral from her general practitioner (GP) due to hypokalaemia. Her past medical history includes postural hypotension, multifactorial falls, hydrocephalus with a ventriculoperitoneal shunt, poor eyesight, and hearing impairment. She lives alone at home, receives a care package three times daily, and mobilises independently at baseline.

The patient reported a gradual onset of tiredness, generalised weakness, and muscle cramps over several days. She also noticed reduced exercise tolerance, struggling to walk distances she would normally manage, and had sustained one recent fall at home without loss of consciousness or head injury. She attended her GP for assessment, where blood tests revealed hypokalaemia, prompting referral to the Accident and Emergency Department. Medication review revealed long-term use of fludrocortisone 100 micrograms once daily for postural hypotension.

There were no symptoms suggestive of infection, chest pain, palpitations, vomiting, or diarrhoea.  Further review of her medical history showed that she had been on fludrocortisone for a prolonged period with regular GP follow-up and urea and electrolytes (U&E) monitoring. She had previously developed asymptomatic mild hypokalaemia, which had been managed in the community with oral potassium supplementation and ongoing monitoring.

On examination, the patient was alert but mildly confused. Cognitive assessment using the 4 ‘A’s Test (4AT) scored 2, as she was unable to name the hospital but was aware she was in a hospital and had difficulty counting backwards. Pupillary assessment was not possible due to a pre-existing ophthalmological condition. Cardiovascular examination revealed normal heart sounds with no murmurs, and respiratory examination demonstrated clear lung fields bilaterally. Abdominal examination showed a soft, non-tender abdomen with no organomegaly. Neurological examination revealed no focal neurological deficits. A post-fall assessment identified no acute injuries. Laboratory investigations are summarised in Table 1.

**Table 1:** Summary of laboratory investigations Hb: haemoglobin; eGFR: estimated glomerular filtration rate; WCC: white cell count

Parameters	Patient values	Reference range
Hb	107 g/L	115-169 g/L
WCC	3.9 ×10^9^/L	4-11 x 10^9^/L
Platelets	184 ×10^9^/L	150-400 x 10^9^/L
Glucose	5.5 mmol/L	3.5-5.5 mmol/L
Urea	5.6 mmol/L	2.5-6.7 mmol/L
Creatinine	79 µmol/L	44-71 µmol/L
eGFR	59 mL/min/1.73 m^2^	>69 mL/min/1.73 m^2^
Sodium	143 mmol/L	133-146 mmol/L
Potassium	2.8 mmol/L	3.5-5.3 mmol/L

**Figure 1:**
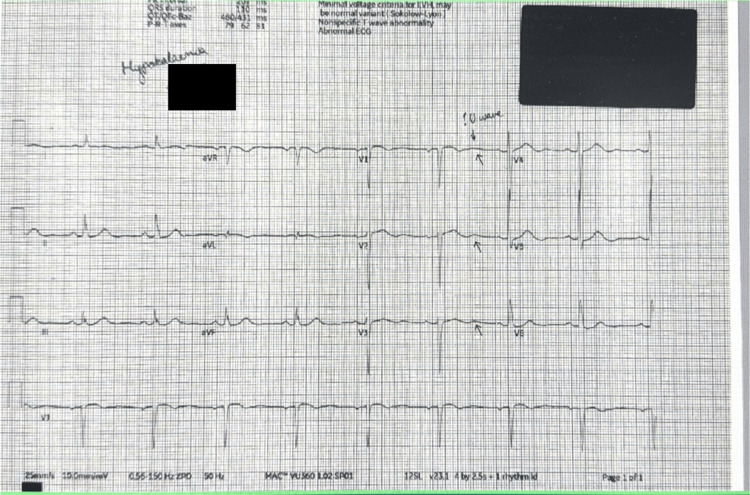
ECG showing normal sinus rhythm with prominent U waves

The ECG findings are a normal sinus rhythm with prominent U waves, consistent with hypokalaemia (Figure 1).

The working diagnosis was symptomatic hypokalaemia secondary to long-term fludrocortisone therapy for the management of postural hypotension.

As initial management, the patient was commenced on intravenous potassium replacement via slow infusion of fluids containing 40 mmol potassium chloride, with cardiac monitoring and was admitted to the medical ward. During her inpatient stay, the dose of fludrocortisone was gradually reduced and tapered over three days. Midodrine 2.5 mg was initiated with an overlap period to manage her postural hypotension. Lying and standing blood pressures were monitored regularly, and serial electrolyte measurements demonstrated a gradual correction of her potassium levels. Her postural symptoms improved following initiation of midodrine therapy. The patient’s potassium levels normalised, and her symptoms of weakness and postural hypotension improved. She was deemed medically stable for discharge. Midodrine therapy was continued, and fludrocortisone was discontinued. Follow-up was arranged with the GP for ongoing blood pressure and electrolyte monitoring.

## Discussion

Hypokalaemia represents one of the most frequently encountered electrolyte disturbances in clinical practice, with particular significance in older adult populations [7]. This case exemplifies the complex nature of hypokalaemia management in elderly patients, where multiple contributing factors necessitate careful and comprehensive clinical assessment.

Fludrocortisone, a synthetic mineralocorticoid, exerts its effects by binding to mineralocorticoid receptors in the distal tubules and collecting ducts of the kidney, where it enhances sodium reabsorption and concurrently increases renal potassium excretion, contributing to hypokalaemia. Its activation of intracellular receptors leads to increased expression of sodium transport proteins such as epithelial sodium channels (ENaC) and Na⁺/K⁺-ATPase pumps, promoting sodium and water retention while facilitating potassium loss. The resulting electrolyte shifts may also influence acid-base balance by enhancing hydrogen ion secretion, thereby contributing to metabolic disturbances associated with mineralocorticoid therapy [8]. In a prospective study of elderly patients treated with fludrocortisone for hypotensive disorders, hypokalaemia developed in a noteworthy proportion of participants, illustrating that even low-dose therapy can be poorly tolerated and contribute to clinically relevant potassium depletion in this population [6].

Importantly, hypokalaemia often develops gradually and may manifest with nonspecific symptoms such as weakness, muscle cramps, or fatigue, which can delay clinical recognition [9]; if uncorrected, the resulting electrolyte imbalance can lead to significant cardiac electrophysiological changes [10], including characteristic ECG findings such as prominent U waves seen in this patient.

In this case, fludrocortisone was identified as the likely driver of renal potassium wasting, reflecting its potent mineralocorticoid activity that has been associated with significant electrolyte disturbances, including hypokalaemia. This case highlights how routine medications can be a simple yet easily missed cause of electrolyte disturbance. In clinical practice, hypokalaemia is often attributed to more common causes such as diuretics, gastrointestinal losses, or poor oral intake [11], which may result in fludrocortisone being overlooked during medication review.

Management in this patient involved discontinuation of fludrocortisone and switching to midodrine, an alternative agent for orthostatic hypotension that does not carry the same risk of electrolyte imbalance [12]. Following this change, potassium levels normalised, supporting a causal relationship. This underscores the importance of reviewing medication necessity and considering safer alternatives in frail or elderly patients.

Therefore, this case serves as a reminder that medication-induced hypokalaemia is a preventable and reversible condition. Regular monitoring of electrolytes [7] and careful medication review are essential, particularly in older patients receiving mineralocorticoids. Awareness of this association can help clinicians avoid unnecessary investigations and reduce the risk of complications associated with severe hypokalaemia.

## Conclusions

This case demonstrates fludrocortisone-induced hypokalaemia as an important and reversible cause of functional decline and falls in an older adult. The non-specific presentation with weakness, confusion, and reduced mobility highlights how electrolyte disturbances can be easily overlooked or attributed to other common geriatric syndromes. Orthostatic hypotension is frequently encountered in older people, and mineralocorticoids such as fludrocortisone are often prescribed due to their perceived safety and efficacy; however, they may occasionally be associated with clinically significant adverse effects, including hypokalaemia. In this case, fludrocortisone was considered a likely contributing factor, although a multifactorial aetiology cannot be excluded. Careful medication review is therefore essential when assessing hypokalaemia in elderly patients. Regular monitoring of serum electrolytes, particularly in those at higher risk, and consideration of alternative treatments such as midodrine may help reduce adverse effects. Increased awareness of medication-related hypokalaemia can assist clinicians in preventing avoidable morbidity and improving outcomes in frail older adults.
